# The Bidirectional Shape Memory Effect of Polyurethane Photocrosslinked with Polycaprolactone and Hexamethylene Diisocyanate

**DOI:** 10.3390/ma19112338

**Published:** 2026-06-01

**Authors:** Qiang Xu, Ziheng Sang, Yanmei Jin, Ze Chen, Chao Ma, Haihui Liu

**Affiliations:** 1 School of Mechanical and Power Engineering, Tianjin Renai College, Tianjin 301636, China; xuqiang202606@163.com; 2 Hebei Industrial Technology Research Institute of Membranes, Cangzhou Institute of Tiangong University, Cangzhou 061000, China; 3 State Key Laboratory of Advanced Separation Membrane Materials, School of Materials Science and Engineering, Tiangong University, Tianjin 300387, China; 4 Tianjin Key Laboratory of Equipment Design and Manufacturing Technology, Tianjin Renai College, Tianjin 301636, China

**Keywords:** polyurethane, UV crosslinking, bidirectional shape memory effect

## Abstract

Shape memory polymers (SMPs) can undergo reversible shape transformations, yet most conventional one-way SMPs recover only a single programmed shape. Reported bidirectional SMPs frequently rely on complex chemistries or continuous external loads or tolerate pronounced losses in mechanical robustness, largely because microphase separation, crystallization and internal stress are difficult to regulate in an integrated fashion. Here, we propose a UV-programmed internal-stress-locking strategy to construct a crosslinked polyurethane (UV-SMPU) that simultaneously achieves high toughness and stable, stress-free bidirectional actuation. Using polycaprolactone (PCL) as the soft segment, hexamethylene diisocyanate (HDI) as the hard segment and triallyl isocyanurate (TAIC) as a photocrosslinker, in-situ UV curing under pre-stretch fixes a tunable three-dimensional network while “freezing” the microphase-separated morphology and pre-oriented internal stress. Covalent crosslinks stabilize PCL crystallites as reversible actuation domains, whereas hydrogen-bonded hard segments provide elastic restoring force; the coordinated regulation of crosslink density, crystallinity and locked-in internal stress enables efficient CIE/MIC-type transitions without compromising mechanical integrity. The optimized UV-SMPU (3 wt% TAIC, 10 min UV) exhibits excellent thermal stability, a rare strength–ductility balance (26.6 MPa tensile strength; ~1700% elongation) and robust bidirectional actuation, with reversible strain stabilizing at 15.73% after six cycles. This work offers a simple, scalable route to tough bidirectional SMPUs and furnishes mechanistic design principles for next-generation adaptive and soft-actuated materials.

## 1. Introduction

Shape memory polymers (SMPs) are a class of smart materials that can recover from a temporary shape to their original shape in response to external stimuli such as temperature, light, pH, or magnetic fields [1,2,3]. Owing to their light weight, large deformability, structural programmability, and tunable stimulus responsiveness, SMPs have attracted considerable attention in soft robotics, flexible actuators, implantable medical devices, and biomimetic artificial muscles [4]. Compared with traditional shape memory alloys or ceramics, SMPs possess greater molecular design flexibility, easier processing, and a wider adjustable range of transition temperatures, making them promising candidates for adaptive and programmable deformation systems [5,6]. However, conventional one-way SMPs generally memorize only one temporary shape and require external force for reprogramming after recovery, which limits their use in repeated actuation, cyclic deformation, and multi-stage shape-changing applications [7,8].

To overcome the limitations of one-way SMPs, bidirectional shape memory polymers have been developed to achieve reversible switching between two shapes during heating–cooling cycles without repeated external programming. For semicrystalline SMPs, this reversible deformation is mainly governed by crystallization-induced elongation (CIE) during cooling and melting-induced contraction (MIC) during heating [9,10]. Therefore, the construction of bidirectional SMPs requires not only a reversible crystallization/melting transition but also a stable internal stress field to guide cyclic deformation. In recent years, chemically crosslinked networks, physically programmed semicrystalline structures, dynamic or supramolecular networks, and multilayer/composite actuator systems have been widely explored [11,12,13]. However, these strategies still face a common challenge: insufficient network stability can lead to stress relaxation, whereas excessive crosslinking or rigid structural design may restrict chain mobility and weaken crystallization-driven reversible deformation. Thus, balancing internal stress fixation, chain mobility, crystallization behavior, and mechanical robustness remains a central issue in developing high-performance bidirectional SMPs.

Semicrystalline thermoplastic polyurethane (TPU) provides a promising platform to address this issue because its segmented structure allows independent regulation of soft-segment crystallization, hard-segment physical crosslinking, microphase separation, and mechanical properties [14,15,16]. By adjusting the chemical composition of the soft segments (e.g., polyester or polyether) and hard segments (e.g., diisocyanates and chain extenders), the glass transition temperature (*T_g_*) or melting temperature (T_m_) of the material can be precisely tailored to meet a range of stimulus–response requirements [17,18]. Libor et al. synthesized a high-strength polyurethane using hexamethylene diisocyanate (HDI) as the hard segment [19]. In comparison to commonly used diphenylmethane diisocyanate (MDI) and toluene diisocyanate (TDI), HDI is aliphatic and free of aromatic rings. Its highly ordered molecular structure enhances the flexibility and crystallinity of the polyurethane. Photo-crosslinking has garnered significant attention in the field of SMPs due to its controllability and efficiency [20,21,22,23]. Hu et al. [24] incorporated a photosensitive anthracene group into the hard segment of thermoplastic polyurethane (TPU), achieving stable shape recovery under high strain (270%). Additionally, adequate chemical crosslinking promotes strain hardening, ensuring the material’s strength and toughness [25,26]. Nevertheless, most reported photo-crosslinked SMPU systems mainly focus on one-way shape recovery or mechanical reinforcement, while the use of UV crosslinking to lock pre-stored internal stress for stable bidirectional actuation remains insufficiently explored.

In this work, polycaprolactone (PCL) was selected as the soft segment, 1,4-butanediol (BDO) as the chain extender, and symmetric hexamethylene diisocyanate (HDI) as the hard segment to construct a polyurethane backbone with favorable crystallization ability and microphase separation potential. On this basis, a three-dimensional chemical crosslinked network was formed by introducing triallyl isocyanurate (TAIC) as the crosslinker and 2,2-dimethoxy-2-phenylacetophenone (DMPA) as the photoinitiator, followed by UV irradiation under a pre-stretched state. This integrated “stretching–UV crosslinking” strategy not only promotes a more ordered and tunable microphase-separated structure between the PCL crystalline domains and the hydrogen-bonded hard segments, but also enables the crosslinked network to effectively “capture” and fix internal stress. As a result, the material simultaneously achieves high strength, high toughness, and a stable bidirectional shape memory effect. Systematic investigations on the effects of soft/hard segment ratio, crosslinker content, and UV exposure time on the microphase structure, thermo-mechanical behavior, and bidirectional shape memory performance reveal the key roles of crosslink density, crystallization behavior, and internal stress regulation in governing reversible two-way deformation. This study provides a new and broadly applicable design concept for constructing high-performance bidirectional shape memory polyurethanes via a simple UV programming route.

## 2. Experimental Section

### 2.1. Materials

Polycaprolactone diol (PCL, Mn = 2000 g mol^−1^) was obtained from the Dingli Plasticizer Additives Department. Before use, PCL was vacuum-dried at 120 °C. 1,4-Butanediol (BDO) and Dibutyltin dilaurate (DBTDL) were available from Tianjin KeMio Chemical Reagent Co., Ltd., Tianjin, China. Hexamethylene diisocyanate (HDI, 99%), triallyl isocyanurate (TAIC, 98%), and 2,2-dimethoxy-2-phenylacetophenone (DMPA) were purchased from Shanghai Macklin Biochemical Technology Co., Ltd., Shanghai, China. N,N-dimethylformamide (DMF) was obtained from Tianjin Fengchuan Chemical Reagent Technology Co., Ltd., Tianjin, China.

### 2.2. Polyurethane Synthesis

**Figure 1:**
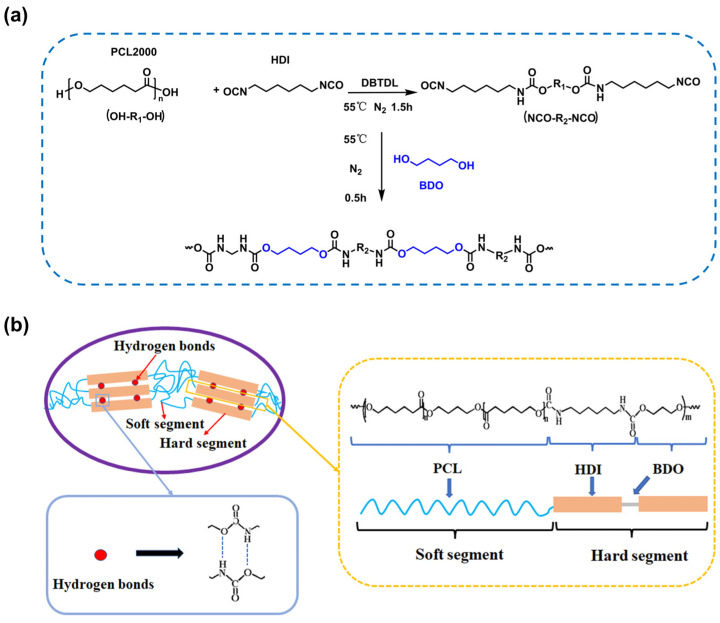
(**a**) TPU reaction equation. (**b**) Microstructure diagram of TPU.

A series of polyurethanes with varying soft segment content were synthesized using a two-step method, following the composition ratios listed in Table S1 of the Supporting Information. PCL was dried in a vacuum oven at 120 °C for 4 h before use. It was then combined with HDI in a three-necked flask, with two drops of DBTDL added as a catalyst, and reacted at 55 °C for 1.5 h. After the prepolymerization reaction was complete, an appropriate amount of the chain extender BDO was added to the system, followed by a reaction at 55 °C for 30 min. During the reaction, DMF was used to adjust the system’s viscosity, and the entire process was carried out under a nitrogen atmosphere. Upon completion of the reaction, the solution was poured into a polytetrafluoroethylene (PTFE) mold and placed in a vacuum oven at 60 °C for 48 h to remove the solvent. Figure 1a,b present the reaction equation and schematic diagram of TPU synthesis.

### 2.3. Preparation and Programming of UV-Crosslinked Polyurethane

Based on the composition ratios listed in Table S2 of the Supporting Information, bidirectional reversible shape memory polyurethanes (UV-SMPUs) with different crosslinker contents and UV irradiation times were prepared through programming and UV-induced crosslinking. TPU-3 was dissolved in a predetermined amount of DMF under mechanical stirring at 60 °C to obtain a 10 wt% TPU solution. Subsequently, the crosslinking agent TAIC and 1 wt% photoinitiator DMPA were added sequentially and stirred thoroughly. The resulting mixture was poured into a polytetrafluoroethylene (PTFE) mold and dried in a vacuum oven at 60 °C for 48 h to remove the solvent. For programming, the sample was heated to 60 °C, above the melting temperature (Tm) of PU, maintained for 30 min, and then stretched to four times its original length. The pre-stretched sample was irradiated with 365 nm UV light at an intensity of 1800 mW/cm^2^, with a distance of 15 cm between the UV light source and the sample, to form stable chemical crosslinking points. After cooling and shape fixation, the UV-SMPU was obtained.

## 3. Results and Discussion

**Figure 2:**
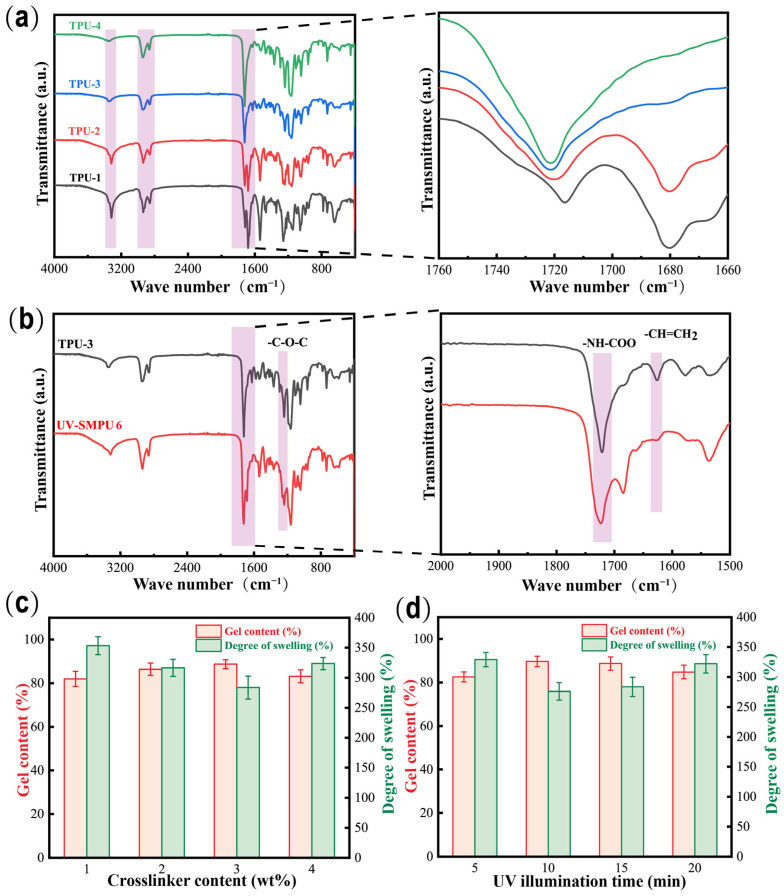
(**a**) FTIR spectra of TPU. (**b**) FTIR spectra of TPU 3 and UV-SMPU 6. Gel content and degree of swelling of UV-SMPU: (**c**) different cross-linking contents; (**d**) different cross-linking times.

TPU and UV-SMPU were characterized by FTIR to analyze their structure. Figure 2a presents the FTIR spectra of TPU synthesized with varying soft segment contents, where the characteristic peaks of functional groups remain largely consistent. The -NCO vibration absorption peak near 2270 cm^−1^ disappears, indicating that the -OH and -NCO groups have completely reacted to form urethane (-NH-CO-) bonds. The peaks at 3340 and 2945 cm^−1^, corresponding to the stretching and bending vibrations of the urethane N−H bond, confirm the formation of PU. The localized FTIR spectra reveal that as the hard segment content increases, the C=O stretching vibration of the carbamate group is observed at approximately 1721 cm^−1^. Simultaneously, the proportion of hydrogen-bonded carbonyl groups rises, while the proportion of free carbonyl groups decreases. The hydrogen bonding between hard segments and the crystallinity of the soft segments contribute to microphase separation [27]. The hard segments aggregate through strong hydrogen bonding to form stable, dispersed regions, which act as physical crosslinkers, while the semicrystalline polycaprolactone (PCL) serves as the primary determinant of the shape-memory behavior of polyurethane [13].

The infrared spectra of TPU-3 and UV-SMPU 6, as shown in Figure 2b, indicate that the peak at 1720 cm^−1^ corresponds to the characteristic absorption of the urethane group in polyurethane, while the peak at 1628 cm^−1^ is attributed to the stretching vibration of the -CH=CH_2_ group. The localized FT-IR spectra on the right side of Figure 2b reveal a distinct small peak prior to crosslinking, and the absorption intensity of the -CH=CH_2_ stretching vibration decreases after crosslinking. This suggests that when active free radicals react with the double bonds of the monomer, the molecular chains grow and become highly crosslinked, forming a crosslinked network polymer [28]. These findings also confirm the successful preparation of UV-SMPU 6.

Gel content can be used to characterize the degree of crosslinking in the material. Figure 2c illustrates that the degree of crosslinking varies with the amount of crosslinking agents. The gel content (*Q_g_*) of the UV-SMPU samples first increases and then decreases as the TAIC content rises, indicating that the degree of crosslinking in the polymer follows a similar trend. At a TAIC mass fraction of 3%, the gel content reaches its maximum value due to the decomposition of DMPA, which generates a higher number of active free radicals. This leads to increased crosslinking efficiency and a greater number of crosslinked structures. However, when the TAIC mass fraction exceeds 3%, the gel content decreases. Excessive TAIC results in an overproduction of free radicals from DMPA decomposition, which can induce degradation and cage effects, increasing the loss of free radicals and subsequently decreasing both gel content and crosslinking efficiency.

The UV exposure time significantly impacts the degree of crosslinking. As illustrated in Figure 2d, the gel content initially increases with longer crosslinking times but then decreases, while the swelling degree exhibits the opposite trend. This occurs because, at the beginning of the reaction, the photolysis rate of the photoinitiator is relatively high. As the reaction progresses, the initiator concentration gradually diminishes. Once the initiator has decomposed to a certain extent, the crosslinking reaction halts. As the crosslinked network develops, the generation of reactive sites becomes suppressed, leading to a slowdown and eventual cessation of the crosslinking process. Consequently, when the photoinitiator content is 3% and the UV exposure time is 10 min, the gel content of UV-SMPU reaches its maximum, along with the highest degree of crosslinking.

**Figure 3:**
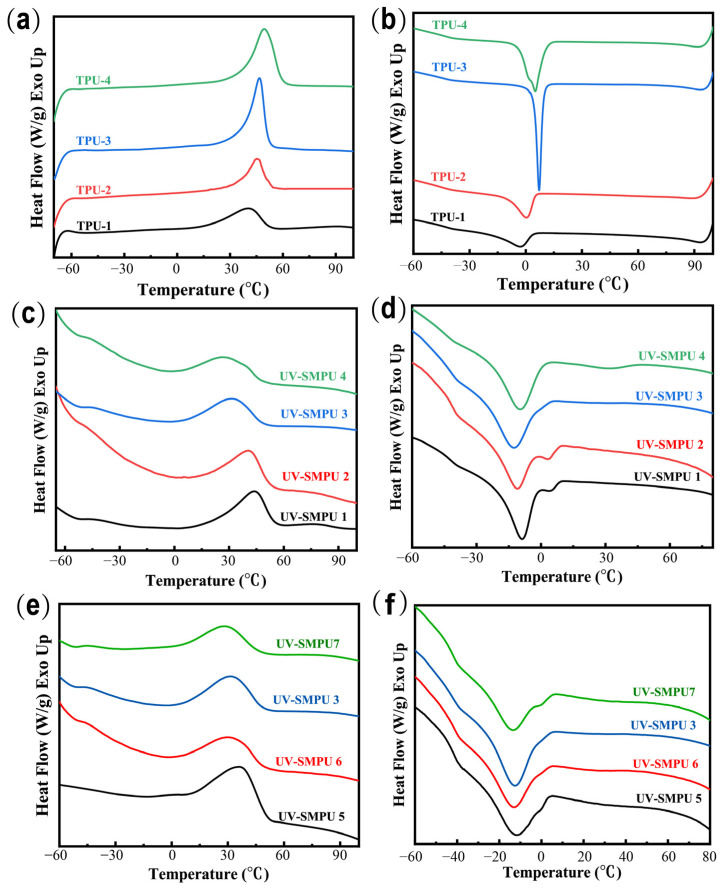
DSC curves of TPU and UV-SMPU: (**a**,**c**,**e**) melting curves and (**b**,**d**,**f**) crystallization curves.

As shown in Figure 3a,b and Table S3, the thermal transition behavior of TPU is closely related to the soft-segment content. With increasing PCL soft-segment content from TPU-1 to TPU-4, the melting temperature (Tm) increases from 40.0 °C to 49.4 °C, and the crystallinity (Xc) increases from 28.65% to 43.87%. The crystallization temperature (Tc) also generally shifts to a higher temperature, from −3.0 °C for TPU-1 to 7.1 °C for TPU-3, although a slight decrease is observed for TPU-4 (5.1 °C). These results indicate that higher PCL content facilitates chain rearrangement and crystal growth, leading to more complete PCL crystalline domains. In contrast, a higher hard-segment content restricts the mobility and regular packing of PCL chains through hydrogen-bonded hard domains and partial hard/soft segment mixing, resulting in lower crystallinity and less perfect crystals [28].

After UV crosslinking, the DSC curves of UV-SMPU show obvious changes in both melting and crystallization behavior, as shown in Figure 3c–f and Table S3. For the samples with increasing TAIC content, Tm decreases from 43.6 °C for UV-SMPU 1 to 28.7 °C for UV-SMPU 4, while Tc shifts from −8.9 °C to −13.7 °C, and Xc decreases from 22.89% to 18.49%. Similarly, with prolonged UV irradiation, Tm decreases from 35.9 °C for UV-SMPU 5 to 28.4 °C for UV-SMPU 7, and Xc decreases from 22.64% to 16.08%. These changes confirm that increasing crosslink density suppresses the crystallization of PCL soft segments. The decrease in Tm suggests the formation of less perfect or thinner PCL crystallites, while the lower Tc indicates that crystallization requires a higher degree of supercooling, reflecting reduced crystallization ability during cooling. The broadened melting and crystallization peaks further imply a broader crystal-size distribution and a more heterogeneous microphase structure [29].

Therefore, UV-induced crosslinking has a dual effect on the UV-SMPU network. On the one hand, the chemical crosslinking points restrict chain mobility and stabilize the programmed network. On the other hand, excessive crosslinking reduces the crystallinity and crystal perfection of PCL, which may weaken crystallization-driven reversible deformation. Thus, appropriate TAIC content and UV irradiation time are essential for balancing network constraint and PCL crystallization, providing the thermal structural basis for bidirectional shape memory behavior [30].

**Figure 4:**
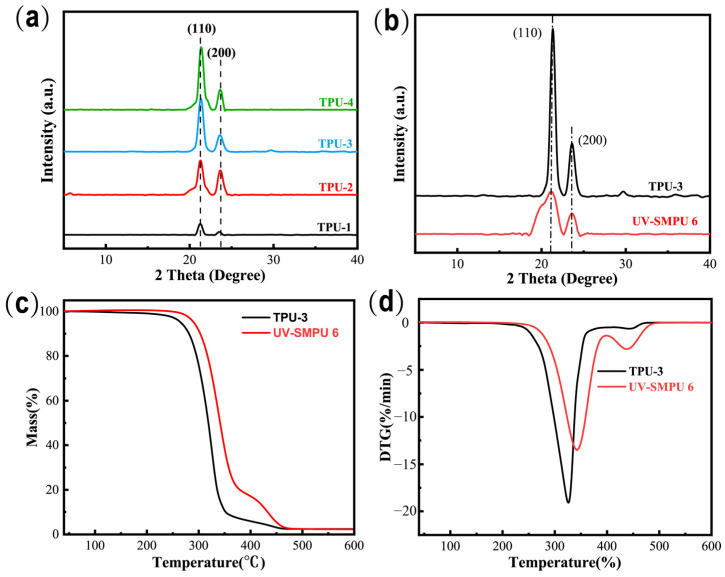
WAXS profiles (**a**) TPU (**b**) TPU 3 and UV-SMPU 6. TG and DTG curves of UV-SMPU 6: (**c**) TG and (**d**) DTG.

As shown in Figure 4a, all TPU samples exhibit two characteristic diffraction peaks at approximately 21.4° and 23.7°, corresponding to the (110) and (200) planes of PCL crystalline domains. The unchanged peak positions indicate that the soft/hard segment ratio does not change the crystal form of PCL. However, the peak intensity gradually increases from TPU-1 to TPU-4, suggesting enhanced PCL crystallization with increasing soft-segment content. This is consistent with the DSC results, where the crystallinity increases from 28.65% for TPU-1 to 43.87% for TPU-4. In contrast, higher hard-segment content restricts PCL chain mobility through hydrogen-bonded hard domains and partial hard/soft segment mixing, thereby suppressing ordered crystal growth.

Figure 4b further shows that UV-SMPU 6 retains the characteristic PCL diffraction peaks after UV crosslinking, indicating that the crystal form of PCL is preserved. Nevertheless, the peak intensity is clearly reduced compared with TPU-3, confirming that the TAIC-based crosslinked network restricts PCL chain rearrangement and reduces crystallinity. Therefore, WAXS results, together with DSC analysis, demonstrate that UV crosslinking mainly weakens the crystallization ability and crystal perfection of PCL rather than changing its crystal structure, which is closely related to the balance between network constraint and reversible crystallization-driven deformation [31].

Figure S1 displays the TGA curves of TPU with varying hard segment contents. As shown, TPU with different hard segment concentrations exhibits a similar mass loss trend as the temperature increases. As the hard segment content increases, the barrier effect of the soft segments becomes more pronounced, hindering the aggregation of hard segments. This reduces the degree of microphase separation and, consequently, decreases thermal stability. When the hard segment content reaches a certain threshold, the barrier effect weakens, leading to an increased degree of microphase separation, which subsequently enhances the material’s thermal stability. Figure 4c,d present the TG and DTG curves of TPU-3 and UV-SMPU 6. After UV crosslinking, the initial decomposition temperature and the maximum thermal decomposition temperature in the first stage of UV-SMPU 6 are higher than those of TPU-3. This is attributed to UV crosslinking forming a three-dimensional network structure that increases intermolecular crosslinking density, thereby improving the thermal stability of TPU.

**Figure 5:**
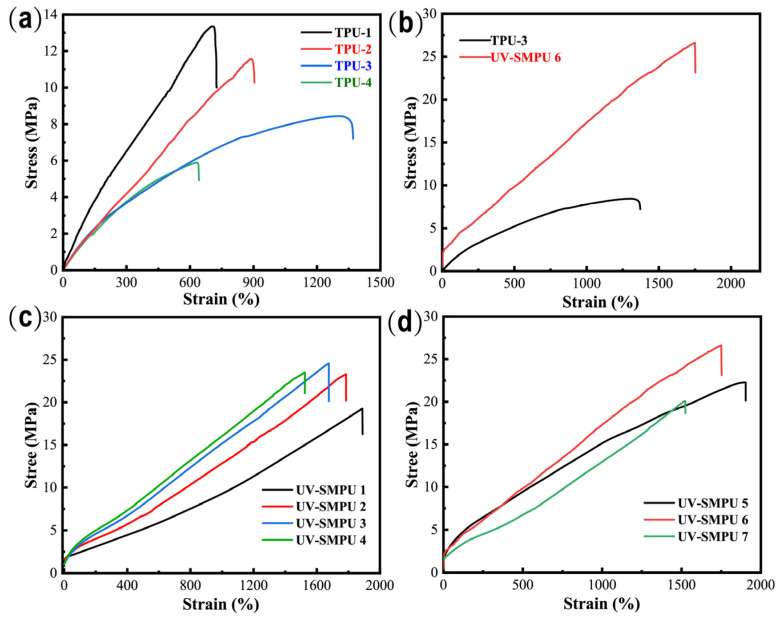
Stress–strain curves of TPU and UV-SMPU. (**a**) TPU (**b**) TPU 3 and UV-SMPU 6. (**c**,**d**) UV-SMPU.

The mechanical properties of TPU with varying hard segment contents are shown in Figure 5a and Table S4. The stress–strain curves of TPU exhibit typical rubber-like behavior. As the hard segment content increases, the tensile strength of TPU also increases. This is due to the longer average hard segment length, which results in a higher number of physical crosslinking points that restrict molecular chain mobility. When subjected to higher stress, the material undergoes deformation, facilitating the movement of molecular chains. The elongation at break of TPU first increases and then decreases with rising hard segment content. At low hard segment content, the degree of microphase separation is low, resulting in reduced toughness. Conversely, at high hard segment content, the hard segments begin to form a continuous phase, increasing the number of crosslinking points. This hinders molecular chain mobility due to strong hydrogen bonding interactions. Consequently, as tensile strength increases, elongation at break decreases. Moderate physical crosslinking enhances intermolecular interactions, improving both tensile strength and elongation at break [32].

The tensile properties of UV-SMPU are illustrated in Figure 5c,d and Table S4. When the UV exposure time is fixed at 15 min, tensile strength initially increases and then decreases as the TAIC crosslinker content rises. During the crosslinking reaction, new chemical bonds form between the UV-SMPU macromolecules, transforming UV-SMPU from a linear structure into a three-dimensional network. This transformation increases its cohesive energy density and enhances tensile strength. However, if the crosslinking density is excessively high, the crosslinked network can obstruct the alignment of molecular chains and hinder crystallization. This exacerbates the uneven distribution of the crosslinked network structure, leading to a more uneven stress distribution and ultimately reducing both tensile strength and elongation at break. These findings align with the results from DSC and gel content tests. Figure 5b presents the mechanical properties of TPU-3 and UV-SMPU 6. After UV crosslinking, a three-dimensional crosslinked network structure is formed. Moderate chemical crosslinking enhances both tensile strength and elongation at break [33].

**Figure 6:**
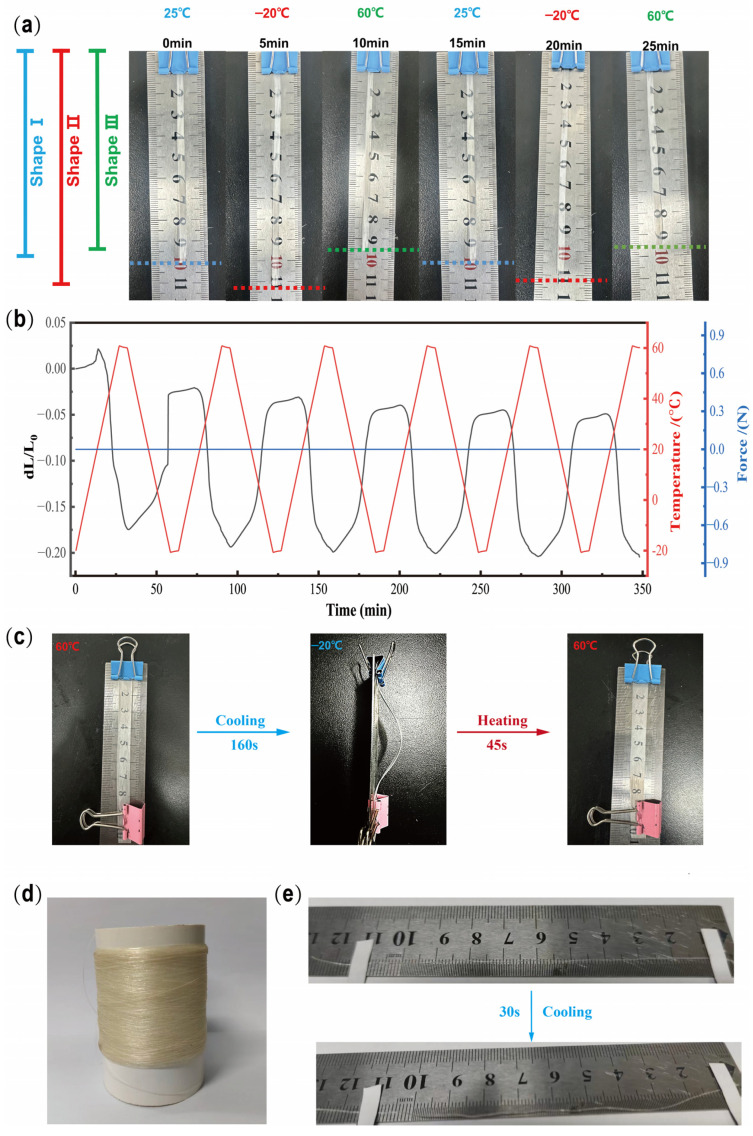
(**a**) Length variation of the programmed UV-SMPU 6 measured during heating–cooling cycles between −20, 25, and 60 °C (**b**) Bidirectional shape memory effect (measured by DMA). (**c**) Reversible bending of a programmed UV-SMPU 6 specimen (clamped at both ends of a steel ruler) after heating–cooling cycles between 60 and −20 °C. (**d**) UV-SMPU 6 fiber. (**e**) The memory effect of UV-SMPU 6 fibers.

After UV crosslinking and programming, the SMPU exhibits stress-free bidirectional shape memory behavior. This reversible actuation originates from the coupling between PCL crystallization/melting and the internal stress fixed by the UV-crosslinked network. During pre-stretching and UV irradiation, the oriented polymer chains are partially locked by newly formed chemical crosslinking points, thereby storing internal stress within the network. When the temperature rises above the melting temperature of the PCL phase, the PCL crystallites melt and chain mobility is restored. The oriented chains then tend to retract under entropic elasticity, resulting in macroscopic contraction. Upon cooling, PCL recrystallization fixes the elongated chain conformation and promotes crystallization-induced elongation, enabling reversible shape transformation without external force, as illustrated in Figure 6a.

To quantitatively evaluate this behavior, UV-SMPU 6 was tested by DMA, as shown in Figure 6b. After eliminating the thermal history in the first heating–cooling cycle, the reversible strain reached 17.22% in the second cycle and gradually stabilized at 15.73% by the sixth cycle. The slight decrease in reversible strain during the initial cycles can be attributed to the relaxation of unstable residual stress and the rearrangement of imperfect PCL crystallites. After repeated thermal cycling, the reversible crystallization/melting of PCL domains and the internal stress fixed by the UV-crosslinked network reach a dynamic balance, resulting in stable stress-free bidirectional shape memory behavior.

As shown in Figure S2, a 3D arch bridge model was designed to evaluate the reversible actuation and recovery behavior of UV-SMPU 6. The sample required 210 s to reach its maximum elongation at −20 °C, whereas only 65 s was needed for recovery at 60 °C. This difference can be attributed to the reduced molecular chain mobility at low temperature, which slows down PCL crystal growth and decreases the crystallization rate, thereby prolonging the crystallization-induced elongation process. In contrast, heating above the melting temperature of PCL rapidly restores chain mobility and accelerates melting-induced contraction. As shown in Figure 6c, the reversible deformation test using a steel ruler further confirms that the SMPU exhibits relatively short deformation and recovery times. These response times are mainly affected by the heating/cooling rate and the thermal conductivity of the material. Higher thermal conductivity facilitates faster heat transfer, allowing the material to reach the target temperature more rapidly and thus accelerating both deformation and recovery processes [34,35].

As shown in Figure S3, UV-SMPU 6 fibers were successfully fabricated through melt-extrusion spinning, programming, and UV crosslinking, and their macroscopic morphology is shown in Figure 6d. The fiber also exhibits an obvious shape memory effect, as demonstrated in Figure 6e, where it elongates effectively under low-temperature conditions. Meanwhile, the high aspect ratio of the fiber promotes rapid heat transfer, endowing it with a faster thermal response. Furthermore, as summarized in Table S5, UV-SMPU 6 exhibits competitive overall performance compared with previously reported two-way shape memory materials, showing both excellent mechanical properties and stable bidirectional shape memory behavior. These results provide useful guidance for the design of polyurethane-based bidirectional shape memory materials and their potential application in thermally responsive actuators.

## 4. Conclusions

In conclusion, we successfully synthesized a UV-crosslinked polyurethane with excellent bidirectional shape memory performance by adjusting the soft–hard segment ratio, crosslinker content, and UV exposure time. UV-SMPU 6 exhibited the highest degree of crosslinking, with a tensile strength of 26.6 MPa and a fracture elongation of approximately 1700%. The UV-crosslinking process provided abundant crosslinking points for the polyurethane, while the crystalline phase of PCL served as a molecular switch. UV-SMPU 6 demonstrated excellent bidirectional reversible shape memory cycling performance under no external stress, stabilizing at 15.73% after six cycles, and exhibited efficient temperature responsiveness. Our work presents a high-strength, controllable, and efficient shape memory polymer, offering a new approach for the application of thermally induced bidirectional reversible SMPUs in the field of smart materials.

## Data Availability

The original contributions presented in this study are included in the article/Supplementary Material. Further inquiries can be directed to the corresponding authors.

## Supplementary Materials

The following supporting information can be downloaded at https://www.mdpi.com/article/10.3390/ma19112338/s1. Figure S1: TG and DTG curves of TPU: (a) TG curve and (b) DTG curve; Figure S2: Reversible bending of a programmed UV-SMPU 6 specimen (fixed at both ends of the paper) after heating–cooling cycles between 60 and −20 °C; Figure S3: UV-SMPU 6 fiber preparation; Table S1: Sample number and composition of TPU; Table S2: Sample number and composition of UV-SMPU; Table S3: DSC data of TPU and UV-SMPU; Table S4: Mechanical properties of TPU and UV-SMPU; Table S5: Comparison of UV-SMPU 6 with reported two-way shape memory materials. Refs. [36,37,38,39,40,41] can be found in Supplementary Materials.
