# Supplementary material for: The Bidirectional Shape Memory Effect of Polyurethane Photocrosslinked with Polycaprolactone and Hexamethylene Diisocyanate
Source: Materials (Basel). 2026 Jun 1;19(11):2338. doi: 10.3390/ma19112338 (PMC13258406; doi:10.3390/ma19112338)
Supplement: Supplementary file 1 [file materials-19-02338-s001.zip › materials-4332660-supplementary.pdf]

Supplementary information

The Bidirectional Shape Memory Effect of Polyurethane Photocrosslinked with Polycaprolactone and Hexamethylene Diisocyanate

1.1 Fourier transform infrared spectroscopy (FTIR)

Infrared absorption spectroscopy testing and analysis were conducted on PU prepolymer, TPU and UV-SMPU using FT-IR with VECTOR22 from Bruker, Germany. The samples were cut into appropriately sized films within the wavelength range of 4000 to 500 cm^{-1}. The test environment was maintained at room temperature, and the analysis was carried out by the functional groups corresponding to the characteristic absorption peaks obtained by FTIR.

1.2 Crystallinity (X_c)

The degree of crystallinity for PCL was calculated from DSC data using equation (1), and for UV-SMPU and TPU, it was determined using the experimental value of ΔH_m relative to the fully crystalline PCL with equation (S2).

$$X_{C1}=\frac{\Delta H_{PCL}}{\Delta H_{O_{PCL}}}\times 100\% \qquad (S1)$$

$$X_{C2}=\frac{\Delta H_{SMPU}}{\Delta H_{O_{PCL}}\times wt.\%}\times 100\% \qquad (S2)$$

where $\Delta H_{O_{PCL}}$ is the enthalpy of melting that can be generated when 100% of PCL chain segments are crystallized, and as reviewed in the literature $\Delta H_{O_{PCL}}$ of PCL is 139.5 J/g and wt.% is the soft segment content in TPU.

1.3 Differential scanning calorimetry (DSC)

The melting and crystallization behaviors of PCL, SMPU, and various stages of SMPU were investigated using a differential scanning calorimeter (DSC204F1, Germany). The measurements were conducted under a nitrogen atmosphere. Temperature cycling was employed for each experiment. For each temperature cycle, the sample was first heated to 100 °C at a rate of 10 °C min^{-1} to eliminate any thermal history. It was then cooled down to -30 °C at the same rate, held at -30 °C for 5 minutes, and finally heated back up to 100 °C at a rate of 10 °C min^{-1}. The entire process was conducted in a nitrogen gas environment.

1.4 X-ray diffraction (XRD)

The crystal structure and degree of crystallinity of the samples were analyzed at room temperature using a BRUKER D8 ADVANCE X-ray diffractometer from Germany fitted with a Cu-Kα radiation source over the range of 2θ = 5-40°.

1.5 Thermogravimetric analysis (TGA)

TGA testing of PCL and different stages of SMPU was carried out using a TG 209 F3 Tarsus thermal weight loss analyzer from NETZSCH Manufacturing GmbH, Germany. The

samples were adequately dried before testing, and the temperature range was 30-600 °C under a N_2 atmosphere with a ramp-up rate of 10 °C min^{-1}.

1.6 Gel Content

The samples were cut into pieces with similar dimensions and extracted in THF at room temperature for 24 h. Subsequently, the samples were dried at 40 °C for 24 h. The gel content and swelling degree were calculated using the original mass of the sample (m_0), the mass of the swollen sample (m_1), and the mass of the dried sample after extraction (m_2), according to Equations (S3) and (S4), respectively.

$$Q_g = \frac{m_2}{m_0} \times 100\% \qquad \text{(S3)}$$

$$Q_s = \frac{m_1}{m_0} \times 100\% \qquad \text{(S4)}$$

1.7 Mechanical properties

A universal testing machine was utilized to measure the tensile strength at break and elongation at break of SMPU materials at different stages. In the tensile test, the tensile speed was 50 mm min^{-1} and the test was conducted at room temperature.

1.8 Shape memory properties

The shape memory properties of SMPU were tested using the METTLER TOLEDO DMA1 manufactured by METTLER TOLEDO International Ltd. in TMA creep mode with N_2 atmosphere; the test temperature range was -20 °C to 60 °C, with a ramp rate of 3 °C min^{-1}. The relationship between temperature, stress, and strain curves was tested using

rectangular sample strips.

The repeatability of the bidirectional reversible shape memory effect can be characterized for SMPU deformation that changes reversibly with temperature. The reversible strain $\Delta\varepsilon$ is calculated by Equation (S5) to characterize the bidirectional reversible shape memory performance:

$$\Delta\varepsilon=\frac{L_C-L_H}{L_0}\times100\% \qquad (S5)$$

where L_C and L_H are the lengths of SMPU at cooling and heating temperature during the driving process, respectively, and L_0 is the initial length of SMPU at room temperature.

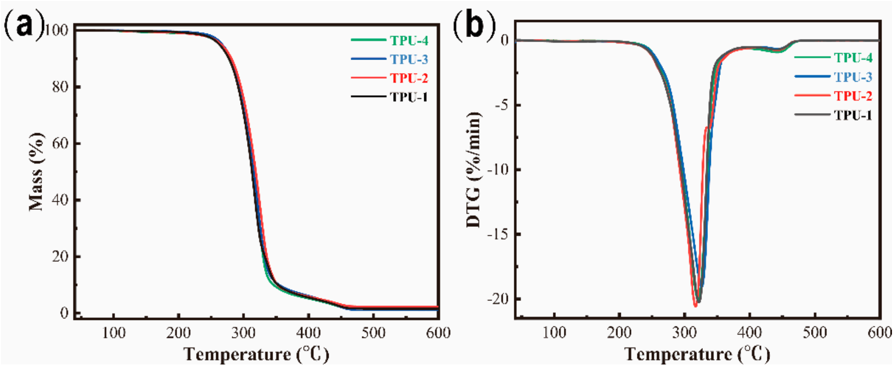

Figure S1 TG and DTG curves of TPU: (a) TG curve and (b) DTG curve.

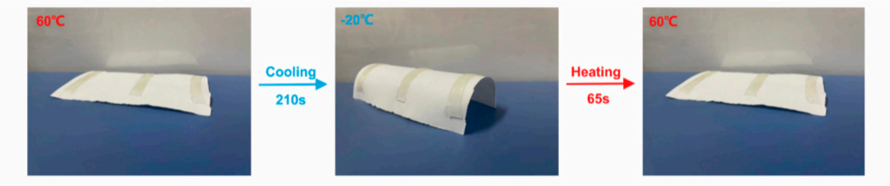

Figure S2 Reversible bending of a programmed UV-SMPU 6 specimen (fixed at both ends of the paper) after heating–cooling cycles between 60 and -20 °C.

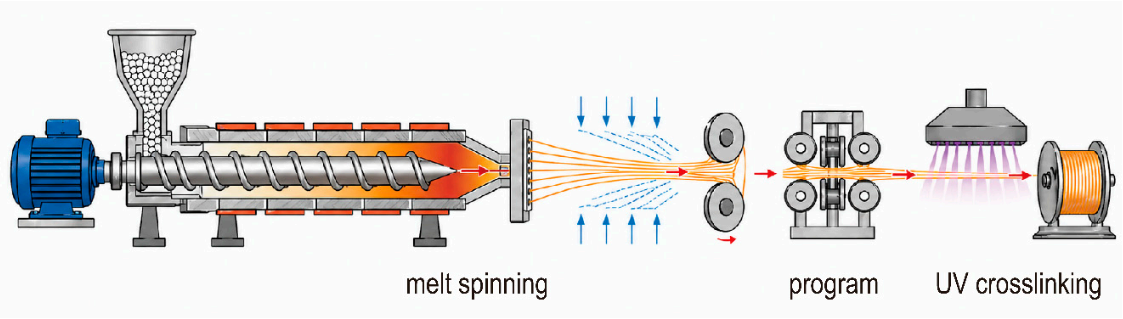

Figure S3 UV-SMPU 6 fiber preparation.

Table S1 Sample number and composition of TPU.

Sample	HDI (mol)	BDO (mol)	PCL_{2k} (mol)	Xc（%）(Soft segment content)
TPU-1	0.040	0.030	0.010	67.8
TPU-2	0.030	0.020	0.010	75.0
TPU-3	0.022	0.012	0.010	80.7
TPU-4	0.015	0.005	0.010	87.0

Table S2 Sample number and composition of UV-SMPU.

Sample	TPU-3 (g)	TAIC (g)	DMPA (g)	TAIC (wt%)	UV light time (min)
UV-SMPU 1	20	0.2	0.2	1	15
UV-SMPU 2	20	0.4	0.2	2	15
UV-SMPU 3	20	0.6	0.2	3	15
UV-SMPU 4	20	0.8	0.2	4	15
UV-SMPU 5	20	0.6	0.2	3	5
UV-SMPU 6	20	0.6	0.2	3	10
UV-SMPU 7	20	0.6	0.2	3	20

Table S3 DSC data of TPU and UV-SMPU.

Sample	Tm/(°C)	Tc/(°C)	ΔHm/(J/g)	ΔHc/(J/g)	Xc/(%)
TPU-1	40.0	-3.0	27.16	27.16	28.65
TPU-2	44.9	0.3	34.69	32.74	31.51
TPU-3	46.8	7.1	45	45	40.3
TPU-4	49.4	5.1	53.28	53.99	43.87
UV-SMPU 1	43.6	-8.9	25.26	22.70	22.89
UV-SMPU 2	41.0	-11.2	24.61	22.67	22.51
UV-SMPU 3	31.7	-12.6	22.12	21.86	20.43
UV-SMPU 4	28.7	-13.7	20.02	18.46	18.49
UV-SMPU 5	35.9	-12.0	24.51	18.56	22.64
UV-SMPU 6	32.4	-13.3	20.24	16.90	18.88
UV-SMPU 7	28.4	-10.0	17.41	20.98	16.08

Table S4 Mechanical properties of TPU and UV-SMPU.

sample	Tensile strength（MPa）	Elongation at break（%）
TPU-1	13.34	712.90
TPU-2	11.51	895.10
TPU-3	8.40	1288.62
TPU-4	5.91	632.42
UV-SMPU 1	19.26	1888.84
UV-SMPU 2	23.31	1785.23
UV-SMPU 3	24.56	1675.84
UV-SMPU 4	23.45	1524.17
UV-SMPU 5	22.27	1902.72
UV-SMPU 6	26.64	1741.91
UV-SMPU 7	20.08	1521.14

Table S5 Comparison of UV-SMPU 6 with reported two-way shape memory materials.

Sample	Tensile strength (Mpa)	Elongation (%)	Reversible variables (%)	References
UV-SMPU 6	26.6	1700	15.73	This Work
SMPU	19.47	815.41	16.1	[1]
EVA	64.46	114.33	9	[2]
PU0.1	24.2	2342	15.15	[3]
T12/CNTs-5	12	278	14.4	[4]
Shape memory liquid crystal elastomer sealant	20.7	322	47	[5]
PEG/PCL semicrystalline network	–	70	12	[6]
PUUA	38	490	14.3	[7]
ZDMA/EVA composite yarn	–	–	8	[8]

References

1 Liu, H.;Li, G.;Zhang, J.;Sang, Z.;Chen, Z.;Xu, Q.;Wang, S., Zhang, X. Synthesis and properties of temperature-responsive shape memory polyurethane with secondary crosslinked network structure based on lpo. Polymer. 2024, 311,127559. https://doi.org/10.1016/j.polymer.2024.127559.

2 Qi, X.;Yang, W.;Yu, L.;Wang, W.;Lu, H.;Wu, Y.;Zhu, S.;Zhu, Y.;Liu, X.;Dong, Y., Fu, Y. Design of ethylene-vinyl acetate copolymer fiber with two-way shape memory effect. Polymers (Basel). 2019, 11(10). https://doi.org/10.3390/polym11101599.

3 Guo, Y.;Chen, Y.;Yu, Q.;Liu, H.;Li, H., Yu, Y. Ultra-tough and stress-free two-way shape memory polyurethane induced by polymer segment "spring". Chemical Engineering Journal. 2023, 470,144212. https://doi.org/10.1016/j.cej.2023.144212.

4 Lu, Y.;Wu, Y.;Wu, J.;Yang, P.;Zhang, Y.;Zhao, W.;Zhang, X.;Cui, Z.;Fu, P.;Pang, X., Liu, M. Electro-induced two-way shape memory thermoplastic polyamide elastomer/carbon nanotubes composites. Journal of Materials Research and Technology. 2024, 29,2062-71. https:// doi.org/10.1016/j.jmrt.2024.01.258.

5 Wang, S.;Jia, Y.;Li, Z.;Gao, Y.;Li, Y.;Chen, Z., Wei, Z. A new pavement crack repair sealant with two-way shape memory effect. Construction and Building Materials. 2024, 436,136816. https://doi.org/10.1016/j.conbuildmat.2024.136816.

6 Inverardi, N.;Toselli, M.;Scalet, G.;Messori, M.;Auricchio, F., Pandini, S. Stress-free two-way shape memory effect of poly(ethylene glycol)/poly(ε-caprolactone) semicrystalline networks. Macromolecules. 2022, 55(19), 8533-47. https://doi.org/10.1021/acs.macromol.2c01064.

7 Li, Y.;Ji, L.;Long, C.;Zhang, Y.;Zhao, W.;Li, X.;Zhang, X.;Cui, Z.;Fu, P.;Pang, X., Liu, M. 4d printable shape memory polyamide elastomer by reactive extrusion. ACS Applied Polymer Materials. 2025, 7(14), 8999-9009. https://doi.org/10.1021/acsapm.5c01008.

8 Qi, X.;Liu, Y.;Dai, H.;Zhu, Y.;Dong, Y.;Fu, S.-Y.;Ni, Q., Fu, Y. Development of high performance two-way shape memory zinc dimethacrylate/ethylene vinyl acetate composite fibers for building flexible yarn actuators. Composites Science and Technology. 2022, 224, 109460.

https://doi.org/10.1016/j.compscitech.2022.109460.
